# Supplementary material for: 64Cu-DOTHA2-PSMA, a Novel PSMA PET Radiotracer for Prostate Cancer with a Long Imaging Time Window
Source: Pharmaceuticals (Basel). 2022 Aug 13;15(8):996. doi: 10.3390/ph15080996 (PMC9412875; doi:10.3390/ph15080996)
Supplement: Supplementary file 1 [file pharmaceuticals-15-00996-s001.zip › pharmaceuticals-1850938-supplementary.pdf]

SUPPLEMENTAL MATERIAL

^{64}Cu-DOTHA$_2$-PSMA, a novel PSMA PET radiotracer for prostate cancer with a long imaging time window

Marie-Christine Milot, Ophélie Bélissant Benesty, Véronique Dumulon-Perreault, Samia Ait-Mohand, Patrick O. Richard, Etienne Rousseau, Brigitte Guérin*

Table of content

Code for cellular assays and dynamic PET statistics. S2

Figure S1. ^{1}H-NMR spectrum of DOTHA$_2$(*O*tBu)$_3$ S2

Figure S2. ^{13}C-NMR spectrum of DOTHA$_2$(*O*tBu)$_3$ S3

Figure S3. HPLC chromatogram of DOTHA$_2$-PSMA S3

Figure S4. ESI-MS spectrum of DOTHA$_2$-PSMA S4

Figure S5. HPLC of ^{nat}Cu-DOTHA$_2$-PSMA S4

Figure S6. ESI-MS of ^{nat}Cu-DOTHA$_2$-PSMA S5

Figure S7. UPLC for A) ^{64}Cu-DOTHA$_2$-PSMA (standard), B) ^{64}Cu(OAc)$_2$ and C) ^{64}Cu-DOTHA$_2$-PSMA in the supernatant fraction after 24 h *ex vivo* incubation in mice plasma S5

Figure S8. Radio-TLC for A) ^{64}Cu-DOTHA$_2$-PSMA, B) ^{64}Cu(OAc)$_2$, C) ^{64}Cu-DOTHA$_2$-PSMA in the plasma supernatant of blood sampled 1h after ^{64}Cu-DOTHA$_2$-PSMA injection in mice S6

Figure S9. Standard (A) and radioactive (B) HPLC chromatogram for ^{68}Ga-PSMA-617 .... S6

Figure S10. *In vivo* stability results after injection of ^{64}Cu-DOTHA$_2$-PSMA in balb/c mice: (A) UPLC for urine collected 1h p.i. of ^{64}Cu-DOTHA$_2$-PSMA and (B) radio-TLC for mice liver extract 2h p.i. of ^{64}Cu-DOTHA$_2$-PSMA S7

Table S1. Cellular uptake and internalization detailed results S8

Table S2. Cellular efflux detailed results S9

Table S3. Linear model for ^{64}Cu-DOTHA$_2$-PSMA and ^{68}Ga-PSMA-617 uptake in LNCaP cells. S10

Table S4. Linear model for ^{64}Cu-DOTHA$_2$-PSMA and ^{68}Ga-PSMA-617 internalization in LNCaP cells S10

Table S5. Linear model for ^{64}Cu-DOTHA$_2$-PSMA and ^{68}Ga-PSMA-617 efflux in LNCaP cells.. S10

Table S6. Balb/c mice biodistribution detailed results S11

Table S7. Detailed results for PET region-of-interest activity for non-blocked injection of ^{64}Cu-DOTHA$_2$-PSMA S12

Table S8. Detailed results for PET region-of-interest activity for injection of ^{64}Cu-DOTHA$_2$-PSMA blocked with ^{nat}Cu-DOTHA$_2$-PSMA S13

Table S9. Linear model for ^{64}Cu-DOTHA$_2$-PSMA tumor uptake in dynamic PET S13

Table S10. Tumor-to-organs of interest ratios for ^{64}Cu-DOTHA$_2$-PSMA PET imaging S14

Table S11. Tumor bearing NRG mice biodistribution detailed results S14

Code for cellular assays and dynamic PET statistics.
To assess statistical significance in cellular assays (^{64}Cu-DOTHA$_2$-PSMA vs. ^{68}Ga-PSMA-617) and dynamic PET (blocked vs. non blocked injection), a linear model in R was used with evaluation of residuals (R x64 4.1.1, libraries: readxl, lme4). A Python script was used to produce graphs to visually analyse the model. Spyder 4 (anaconda3) was used as well as a Python 3.8 program.

```
#R pseudo-code
Model = lm("uptake ~ time + radiotracer")
Summary(model)

#add to the data the predictions from each model and residuals when available
datap = cbind(data, prediction = predict(model),
              prediction_residuals = model$residuals)
```

Figure S1. ^{1}H-NMR spectrum of DOTHA$_2$(*O*tBu)$_3$

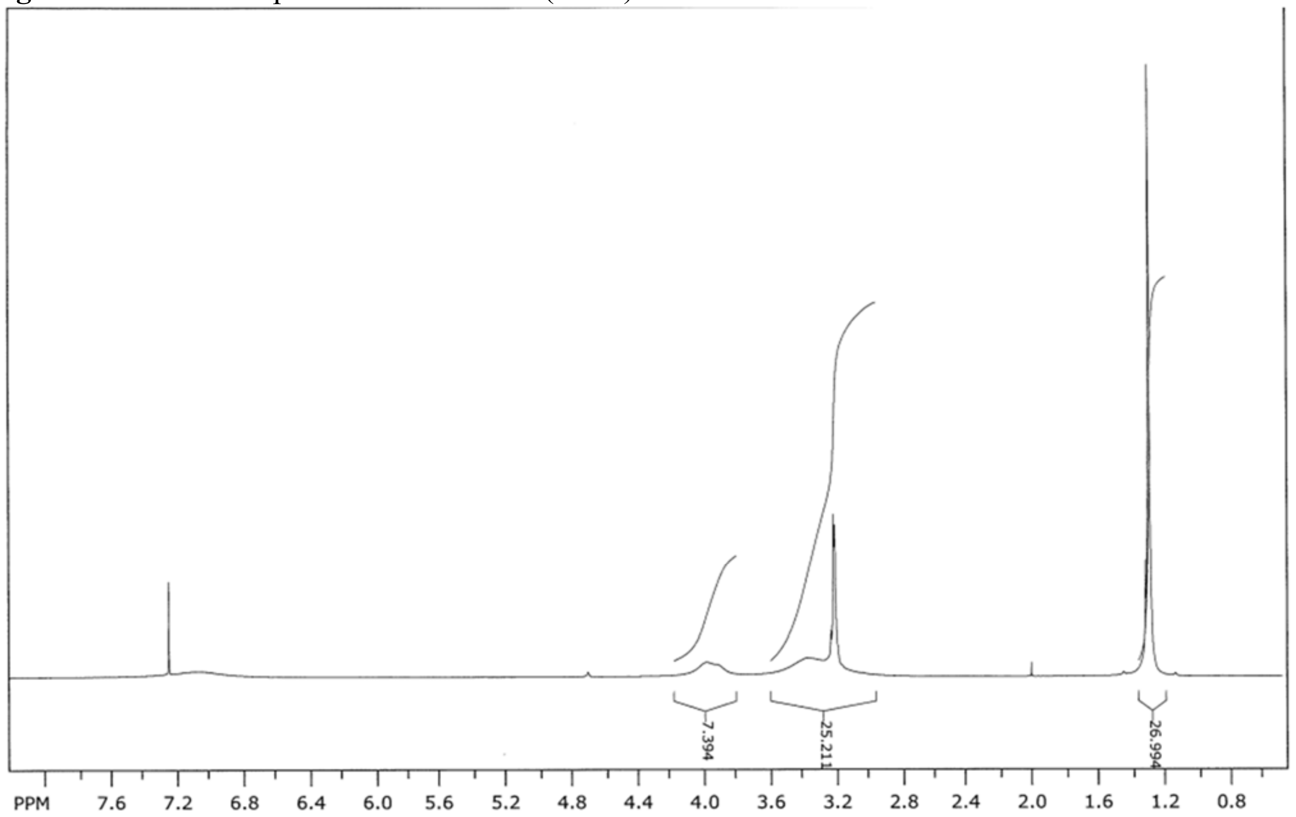

Figure S2. ^{13}C-NMR spectrum of DOTHA$_2$(OtBu)$_3$

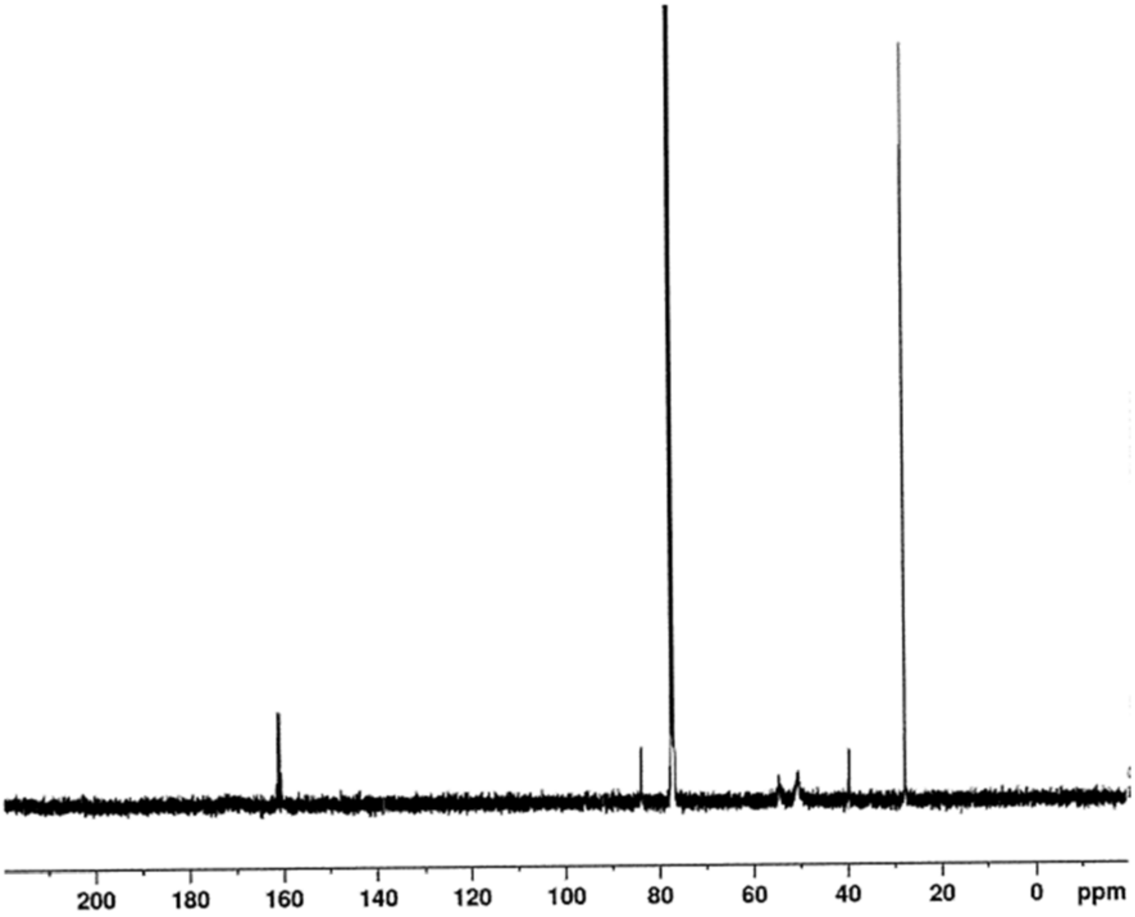

Figure S3. HPLC chromatogram of DOTHA$_2$-PSMA

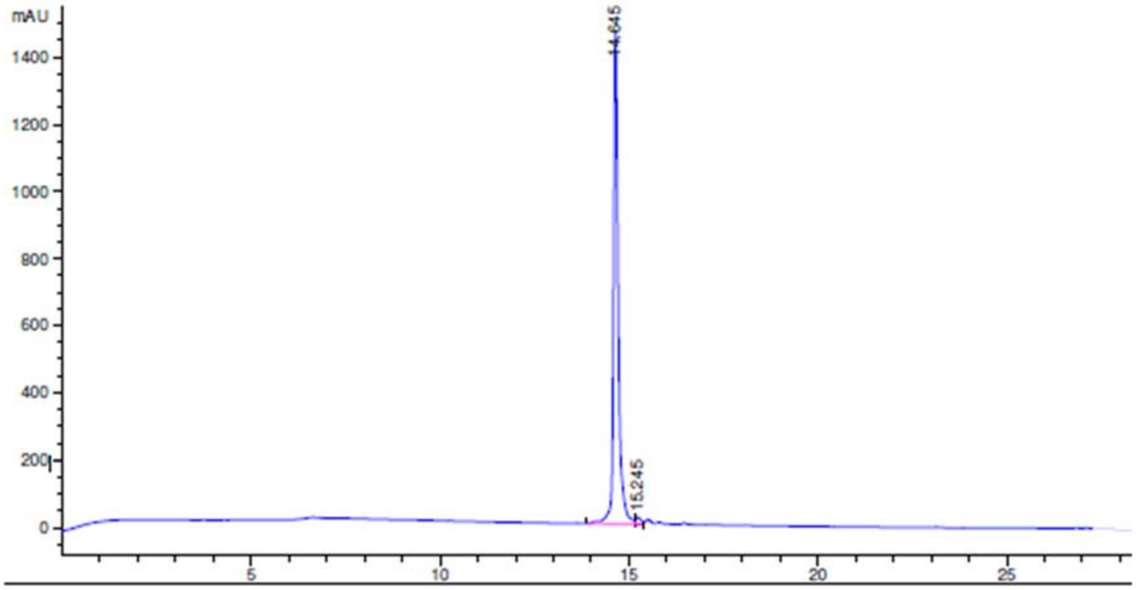

Figure S4. ESI-MS spectrum of DOTHA$_2$-PSMA

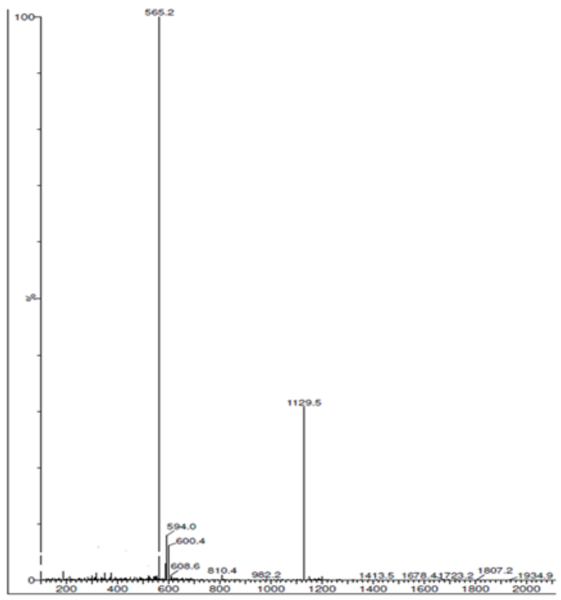

Figure S5. HPLC of ^{nat}Cu-DOTHA$_2$-PSMA

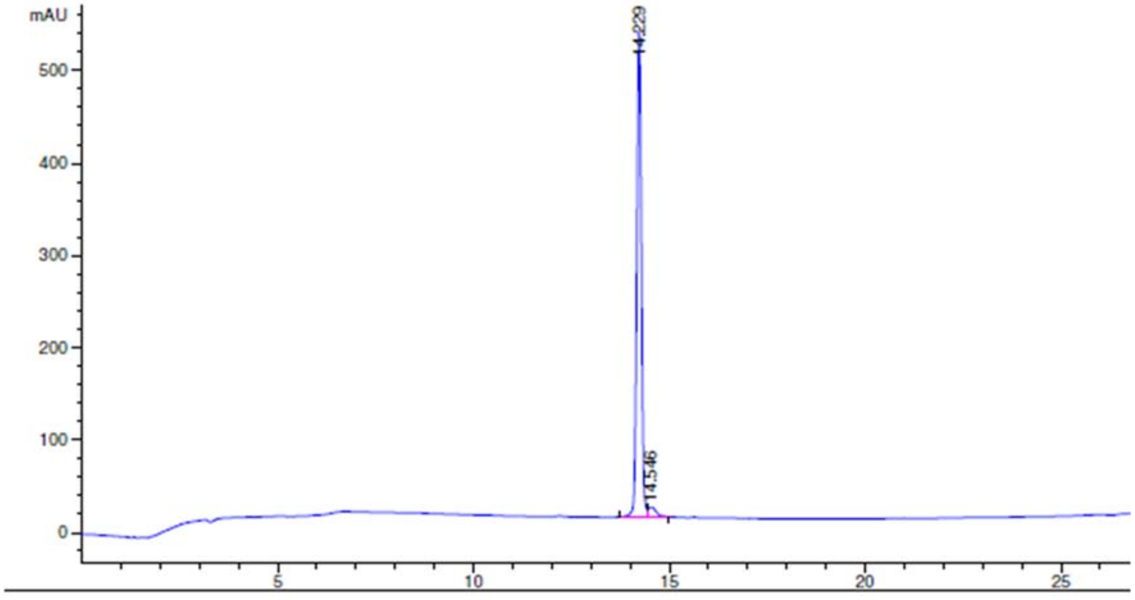

Figure S6. ESI-MS of ^{nat}Cu-DOTHA$_2$-PSMA

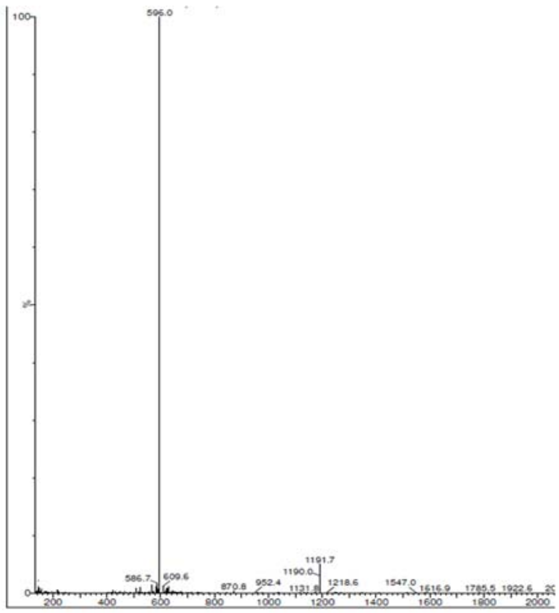

Figure S7. UPLC for A) ^{64}Cu-DOTHA$_2$-PSMA (standard), B) $^{64}Cu(OAc)_2$ and C) ^{64}Cu-DOTHA$_2$-PSMA in the supernatant fraction after 24 h *ex vivo* incubation in mice plasma

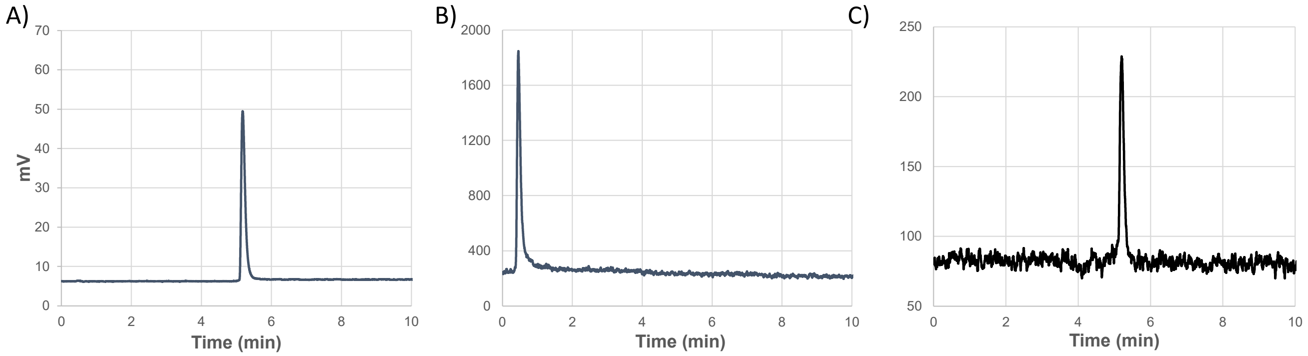

In C, ^{64}Cu-DOTHA$_2$-PSMA was incubated in mouse plasma for 24h and the plasma supernatant was analyses by UPLC. In the plasma supernatant, less than 3% of the signal was free ^{64}Cu, which corresponded to 1.28% of the total plasmatic signal (supernatant plus pellet).

Figure S8. Radio-TLC for A) ^{64}Cu-DOTHA$_2$-PSMA, B) ^{64}Cu(OAc)$_2$, C) ^{64}Cu-DOTHA$_2$-PSMA in the plasma supernatant of blood sampled 1h after ^{64}Cu-DOTHA$_2$-PSMA injection in mice

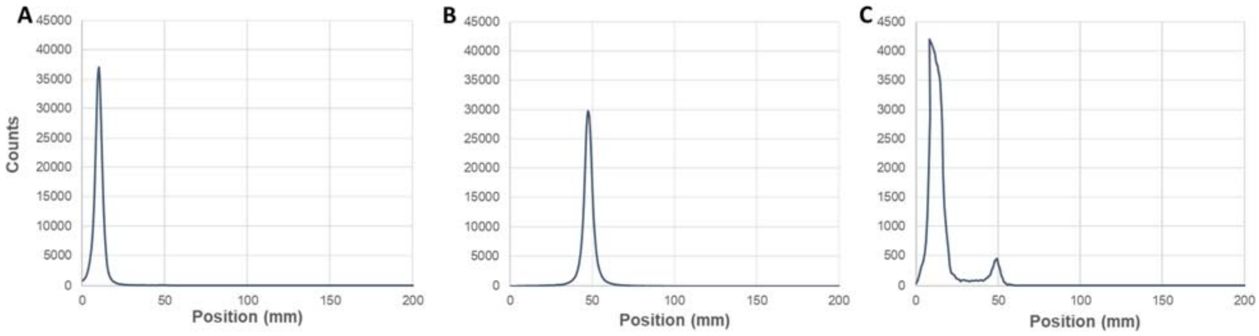

In C, mice were injected with ^{64}Cu-DOTHA$_2$-PSMA and plasma was sampled 1h after the injection to be analyzed by radio-TLC. The plasma supernatant showed a 5% peak from free ^{64}Cu, which corresponded to 2.60% of the total plasma signal (supernatant plus pellet). A larger peak is observed for ^{64}Cu-DOTHA$_2$-PSMA, which may correspond to the tracer alone and bound to plasma protein.

Figure S9. Standard (A) and radioactive (B) HPLC chromatogram for ^{68}Ga-PSMA-617

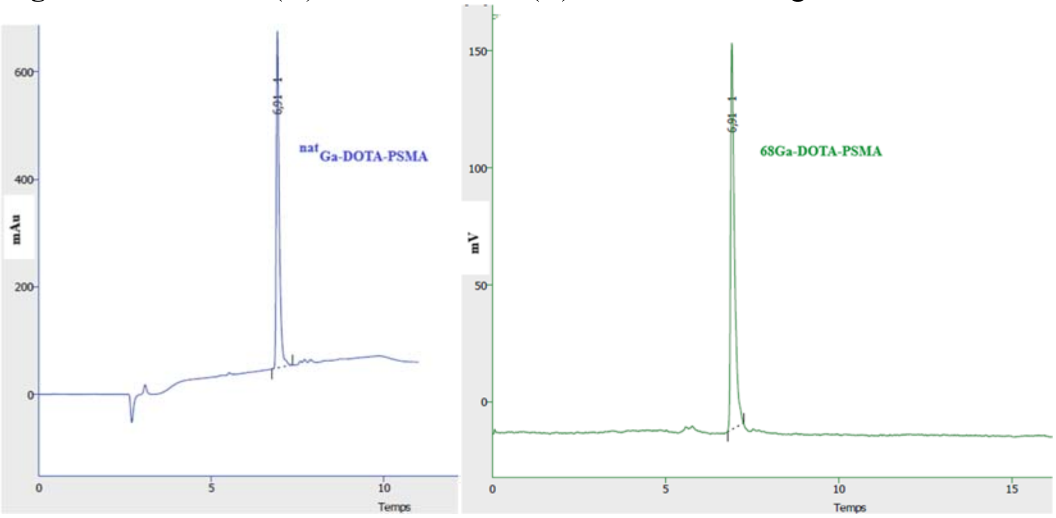

Figure S10. *In vivo* stability results after injection of ^{64}Cu-DOTHA$_2$-PSMA in balb/c mice: (A) UPLC for urine collected 1h p.i. of ^{64}Cu-DOTHA$_2$-PSMA and (B) radio-TLC for mice liver extract 2h p.i. of ^{64}Cu-DOTHA$_2$-PSMA

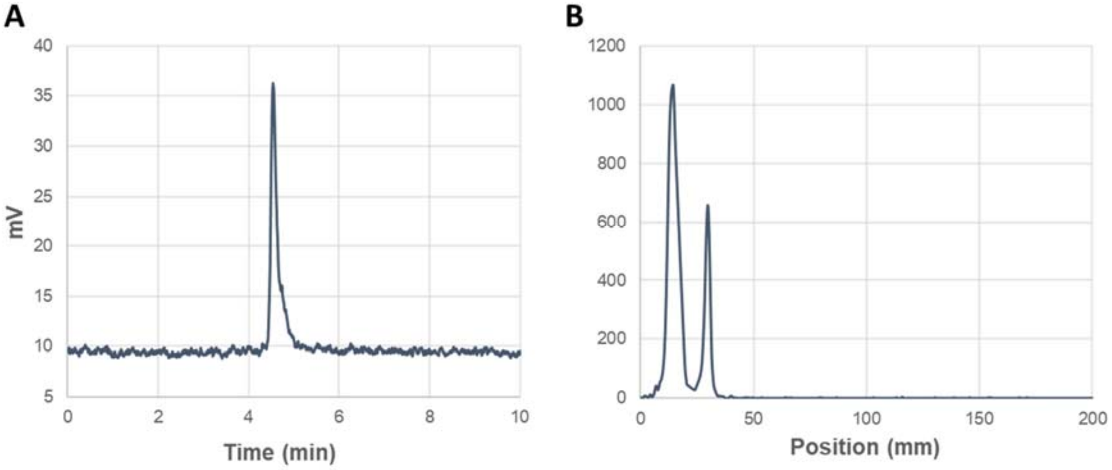

Table S1. Cellular uptake and internalization detailed results

Time p.i. (h)	Uptake								Internalization							
	^{64}Cu-DOTHA$_2$-PSMA Uptake (%IA/10^6 cells)			n	^{68}Ga-PSMA-617 Uptake (%IA/10^6 cells)			n	^{64}Cu-DOTHA$_2$-PSMA Internalized activity (%IA/10^6 cells)			n	^{68}Ga-PSMA-617 Internalized activity (%IA/10^6 cells)			n
0.25	15.39	±	9.57	5	0.00	±	0.00	3	1.68	±	1.04	4	0.00	±	0.00	3
0.5	25.09	±	14.65	5	5.92	±	2.20	3	2.07	±	0.64	4	1.02	±	0.40	3
0.75	22.79	±	14.06	5	8.50	±	2.31	3	2.34	±	0.51	4	1.81	±	0.95	3
1	24.73	±	17.29	5	9.44	±	2.73	3	2.61	±	0.99	4	2.92	±	1.49	3
2	30.24	±	9.65	5	11.44	±	3.14	3	12.25	±	6.69	4	4.38	±	2.05	3
3	32.70	±	11.85	5	13.20	±	2.18	3	14.84	±	5.86	4	7.90	±	2.21	3
4	27.57	±	14.04	9					14.18	±	6.51	6				
8	21.98	±	8.79	4					13.10	±	4.62	2				
12	23.39	±	9.45	4					15.66	±	4.76	2				
24	25.66	±	12.08	4					16.45	±	6.97	2				
36	29.62	±	14.01	4					25.43	±	1.53	2				
48	34.48	±	13.60	4					34.13	±	4.87	2				

Table S2. Cellular efflux detailed results

	Efflux							
	^{64}Cu-DOTHA$_2$-PSMA Retained activity normalized to time 0 (%)			n	^{68}Ga-PSMA-617 Retained activity normalized to time 0 (%)			n
Time p.i. (h)								
1h incubation								
0	100%				100%			
0.25	100.93	±	9.46	4	101.57	±	3.64	3
0.50	105.51	±	6.55	5	106.07	±	13.23	3
0.75	94.38	±	20.10	5	97.22	±	13.20	3
1	77.52	±	39.73	5	84.75	±	28.12	3
2	80.95	±	34.22	5	81.62	±	20.16	3
3	76.53	±	30.57	5				
4	64.04	±	32.06	5				
4h incubation								
4	66.23	±	15.08	4				
8	51.88	±	11.85	4				
24	35.10	±	13.51	4				
32	34.27	±	5.14	4				
48	48.61	±	6.72	4				

Table S3. Linear model for ^{64}Cu-DOTHA$_2$-PSMA and ^{68}Ga-PSMA-617 uptake in LNCaP cells

Predictors	Estimate value of coefficient (95% CI)	T statistic	P value
Effect of time	4.18 (2.59 - 5.77)	5.20	< 0.001
Injecting ^{68}Ga-PSMA-617 instead of ^{64}Cu-DOTHA$_2$-PSMA	-13.82 (-17.93 - -9.12)	-6.65	< 0.001

Adjusted R^2 = 0.397

95% confidence interval (CI) are estimated for coefficient of predictors based on the model degrees of freedom and coefficient standard error.

Table S4. Linear model for ^{64}Cu-DOTHA$_2$-PSMA and ^{68}Ga-PSMA-617 internalization in LNCaP cells

Predictors	Estimate value of coefficient (95% CI)	T statistic	P value
Effect of time	4.52 (3.96 - 5.08)	16.02	< 0.001
Injecting ^{68}Ga-PSMA-617 instead of ^{64}Cu-DOTHA$_2$-PSMA	-0.56 (-1.93 - 0.81)	-0.81	0.42

Adjusted R^2 = 0.693

95% confidence interval (CI) are estimated for coefficient of predictors based on the model degrees of freedom and coefficient standard error.

Table S5. Linear model for ^{64}Cu-DOTHA$_2$-PSMA and ^{68}Ga-PSMA-617 efflux in LNCaP cells

Predictors	Estimate value of coefficient (95% CI)	T statistic	P value
Effect of time	-9.17 (-13.55 - -4.79)	-4.20	< 0.001
Injecting ^{68}Ga-PSMA-617 instead of ^{64}Cu-DOTHA$_2$-PSMA	0.41 (-13.42 - 14.42)	0.06	0.953

Adjusted R^2 = 0.235

95% confidence interval (CI) are estimated for coefficient of predictors based on the model degrees of freedom and coefficient standard error.

Table S6. Balb/c mice biodistribution detailed results

	^{64}Cu-DOTHA$_2$-PSMA					^{68}Ga-PSMA-617	
Organs	1h p.i. (n=9)	2h p.i. (n=6)	4h p.i. (n=6)	24h p.i. (n=5)	48h p.i. (n=5)	1h p.i. (n=4)	2h p.i. (n=4)
Blood	1.47 ± 1.41	1.32 ± 0.47	1.54 ± 0.41	2.09 ± 1.02	1.10 ± 0.23	3.27 ± 2.03	2.12 ± 1.70
Plasma	2.50 ± 1.16	2.60 ± 0.26	3.05 ± 1.18	2.36 ± 0.31	1.57 ± 0.40	4.94 ± 3.50	1.43 ± 0.37
Adrenals	2.13 ± 0.95	3.77 ± 1.27	2.13 ± 1.79	3.00 ± 1.44	2.24 ± 1.03	3.49 ± 0.92	2.19 ± 0.77
Testis	0.65 ± 0.34	1.10 ± 0.11	1.20 ± 0.17	1.35 ± 0.20	0.87 ± 0.13	-	-
Seminal gl.	0.79 ± 0.61	0.71 ± 0.14	0.65 ± 0.16	0.60 ± 0.10	0.51 ± 0.14	-	-
Fat	0.52 ± 0.36	0.51 ± 0.16	0.55 ± 0.24	0.69 ± 0.29	0.38 ± 0.10	0.76 ± 0.16	0.29 ± 0.08
Kidneys	15.76 ± 7.02	9.07 ± 1.24	7.95 ± 1.23	8.63 ± 1.27	5.25 ± 0.75	93.74 ± 26.50	32.38 ± 9.52
Spleen	2.45 ± 1.71	4.55 ± 2.08	3.39 ± 0.82	4.00 ± 0.46	1.76 ± 0.14	2.90 ± 1.20	1.16 ± 0.34
Pancreas	1.42 ± 1.13	1.86 ± 0.34	1.89 ± 0.16	1.92 ± 0.19	1.43 ± 0.36	1.04 ± 0.59	0.36 ± 0.09
Liver	14.27 ± 12.34	29.87 ± 4.98	30.18 ± 4.24	26.91 ± 5.12	13.74 ± 1.56	2.00 ± 1.18	0.67 ± 0.16
Heart	2.72 ± 2.60	3.63 ± 0.49	4.30 ± 0.18	4.43 ± 0.30	3.69 ± 0.78	1.06 ± 0.66	0.28 ± 0.09
Lungs	4.83 ± 3.71	10.44 ± 2.74	8.79 ± 0.44	7.14 ± 0.86	3.76 ± 0.43	3.57 ± 2.41	1.76 ± 0.76
Muscles	0.70 ± 0.64	0.71 ± 0.22	0.65 ± 0.09	0.84 ± 0.07	0.75 ± 0.23	0.57 ± 0.31	0.19 ± 0.06
Bone	0.91 ± 0.63	1.62 ± 0.69	2.32 ± 1.37	1.30 ± 0.18	0.68 ± 0.14	0.52 ± 0.38	0.14 ± 0.04
Brain	0.19 ± 0.15	0.28 ± 0.04	0.32 ± 0.03	0.47 ± 0.07	0.47 ± 0.07	0.11 ± 0.06	0.04 ± 0.01
Tail	3.77 ± 2.49	2.24 ± 0.50	2.37 ± 0.57	2.19 ± 1.25	0.85 ± 0.25	2.69 ± 0.93	2.78 ± 1.60
Intestin	5.71 ± 4.78	11.27 ± 1.66	11.02 ± 0.65	6.29 ± 1.84	2.67 ± 0.52	-	-
Stomach	5.72 ± 5.10	10.58 ± 1.53	9.40 ± 0.91	4.79 ± 0.79	2.82 ± 0.52	-	-
Thyroid	2.93 ± 1.87	3.48 ± 0.67	4.57 ± 3.46	3.18 ± 2.26	1.51 ± 0.58	-	-
Salivary gl.	3.38 ± 2.96	4.40 ± 0.84	3.75 ± 0.30	3.27 ± 0.53	2.13 ± 0.49	-	-
Skin	1.26 0.58	1.56 0.33	1.35 0.61	0.93 0.37	0.39 0.03	-	-

Table S7. Detailed results for PET region-of-interest activity for non-blocked injection of ^{64}Cu-DOTHA$_2$-PSMA

Organs	Tumor	Kidney calyxes	Kidney cortex	Liver	Muscle
0-5 min	6.80 ± 1.38	45.15 ± 23.84	22.79 ± 6.86	12.94 ± 3.68	2.00 ± 0.56
5-10 min	9.29 ± 2.41	49.58 ± 27.77	18.32 ± 5.68	17.82 ± 6.23	2.24 ± 0.66
10-15 min	10.23 ± 3.40	36.82 ± 28.07	15.39 ± 6.00	19.33 ± 7.24	1.62 ± 0.59
15-20 min	10.98 ± 3.50	25.36 ± 17.93	14.02 ± 7.92	20.34 ± 8.34	1.57 ± 0.39
20-25 min	11.62 ± 3.87	21.49 ± 16.94	12.89 ± 8.12	20.86 ± 8.84	1.45 ± 0.53
25-30 min	12.17 ± 4.26	18.56 ± 16.93	12.69 ± 8.69	21.29 ± 9.02	1.68 ± 0.87
30-35 min	12.31 ± 4.35	17.22 ± 17.35	12.25 ± 8.62	21.48 ± 9.10	1.38 ± 0.32
35-40 min	12.83 ± 4.78	16.10 ± 16.00	11.88 ± 8.15	21.85 ± 9.35	1.31 ± 0.44
40-45 min	12.98 ± 4.72	14.37 ± 14.62	11.56 ± 7.82	21.91 ± 9.27	1.34 ± 0.41
45-50 min	13.24 ± 4.94	13.38 ± 13.18	10.53 ± 7.08	22.12 ± 9.35	1.21 ± 0.34
50-55 min	13.90 ± 5.14	12.61 ± 12.19	10.44 ± 6.85	22.47 ± 9.37	1.01 ± 0.35
55-60 min	13.96 ± 4.95	11.28 ± 10.84	9.65 ± 6.34	22.42 ± 9.19	1.09 ± 0.36
4 h	23.79 ± 11.46	-	-	26.85 ± 5.35	1.11 ± 0.29
24 h	18.55 ± 6.56	-	-	21.34 ± 3.59	1.56 ± 0.54

0-1h p.i. non-blocked: n=9, t=14; 4h and 24h p.i. non-blocked: n=6, t=9.

Table S8. Detailed results for PET region-of-interest activity for injection of ^{64}Cu-DOTHA$_2$-PSMA blocked with ^{nat}Cu-DOTHA$_2$-PSMA

	Tumor	Kidney calyxes	Kidney cortex	Liver	Muscle
0-5 min	4.08 ± 1.32	61.33 ± 13.19	18.43 ± 1.48	10.48 ± 0.87	1.58 ± 0.49
5-10 min	4.42 ± 1.19	25.22 ± 4.78	9.65 ± 0.85	15.12 ± 1.01	1.34 ± 0.31
10-15 min	4.50 ± 1.03	16.22 ± 5.12	7.69 ± 1.04	17.33 ± 1.04	0.87 ± 0.05
15-20 min	4.33 ± 0.97	10.32 ± 1.40	6.95 ± 1.28	18.66 ± 1.38	1.06 ± 0.17
20-25 min	4.66 ± 1.01	7.14 ± 1.59	6.51 ± 0.87	18.84 ± 1.07	0.83 ± 0.35
25-30 min	4.30 ± 0.89	6.64 ± 2.11	6.29 ± 1.25	19.42 ± 1.21	0.74 ± 0.18
30-35 min	4.43 ± 1.05	6.53 ± 1.99	6.30 ± 0.92	19.80 ± 1.62	0.69 ± 0.17
35-40 min	4.62 ± 1.16	5.81 ± 1.62	5.52 ± 0.60	19.82 ± 1.44	1.08 ± 0.48
40-45 min	4.68 ± 1.25	6.38 ± 2.05	5.71 ± 0.54	20.31 ± 1.86	0.78 ± 0.28
45-50 min	4.73 ± 1.06	5.18 ± 1.03	6.12 ± 1.32	20.16 ± 1.42	1.14 ± 0.34
50-55 min	4.80 ± 0.99	5.20 ± 1.29	5.58 ± 0.76	20.68 ± 1.58	0.81 ± 0.30
55-60 min	4.79 ± 0.96	5.22 ± 1.53	5.83 ± 0.88	20.29 ± 1.72	0.83 ± 0.21
4 h	7.46 ± 1.08	-	-	19.95 ± 3.11	0.97 ± 0.14
24 h	8.62 ± 0.74	-	-	11.07 ± 1.89	0.65 ± 0.10

n=3, t=6

Table S9. Linear model for ^{64}Cu-DOTHA$_2$-PSMA tumor uptake in dynamic PET

Predictors	Estimate value of coefficient (95% CI)	T statistic	P value
Effect of time	0.08 (-0.18 - 0.34)	5.98	< 0.001
Non blocked injection	7.17 (6.19 - 8.15)	14.41	< 0.001

Adjusted R^2 = 0.503

95% confidence interval (CI) are estimated for coefficient of predictors based on the model degrees of freedom and coefficient standard error.

Table S10. Tumor-to-organs of interest ratios for ^{64}Cu-DOTHA$_2$-PSMA PET imaging

Ratios	1h p.i.	4h p.i.	24h p.i.
Tumor-to-muscle	12.78 (8.58 – 19.26)	21.34 (12.10 – 35.43)	11.86 (7.15 – 20.16)
Tumor-to-kidney cortex	1.45 (0.82 – 3.00)	-	-
Tumor-to-liver	0.62 (0.40 – 1.01)	0.89 (0.52 – 1.24)	0.87 (0.59 – 1.24)

95% confidence intervals on region-of-interest signals were used to calculate the intervals on ratios.

Table S11. Tumor bearing NRG mice biodistribution detailed results

Organs	Non-blocked (n=11)	Blocked (n=4)
Blood	3.46 ± 1.61	1.91 ± 0.30
Plasma	4.52 ± 1.99	2.54 ± 0.14
Adrenals	4.70 ± 3.15	9.02 ± 11.94
Testis	1.96 ± 0.68	1.03 ± 0.10
Seminal gl.	1.13 ± 0.49	0.59 ± 0.05
Fat	0.63 ± 0.23	0.26 ± 0.02
Kidneys	13.24 ± 4.92	6.86 ± 0.20
Spleen	10.10 ± 6.38	6.99 ± 3.36
Pancreas	2.80 ± 0.89	2.09 ± 0.60
Liver	28.54 ± 12.44	15.15 ± 2.93
Heart	5.20 ± 2.05	3.00 ± 0.18
Lungs	14.30 ± 5.78	5.99 ± 0.15
Muscles	1.10 ± 0.56	0.47 ± 0.05
Bone	2.38 ± 2.01	0.91 ± 0.11
Brain	0.75 ± 0.31	0.38 ± 0.03
Tail	2.37 ± 0.87	1.36 ± 0.20
Intestin	15.69 ± 7.36	8.53 ± 1.04
Stomach	9.17 ± 3.26	5.55 ± 0.19
Thyroid	2.57 ± 0.91	1.33 ± 0.28
Salivary gl.	4.18 ± 1.78	1.84 ± 0.05
Skin	1.07 ± 0.54	0.50 ± 0.18
LNCaP tumor	17.33 ± 8.37	7.44 ± 2.31

* Variation on ratios is minimum and maximum calculated from 95% confidence interval.
